# Association of Monoamine Oxidase A Gene Promoter Region (30 bp μVNTR) Polymorphism with Serum Levels in Multiple Psychiatric Disorders

**DOI:** 10.3390/biomedicines13030698

**Published:** 2025-03-12

**Authors:** Aisha Nasir Hashmi, Rizwan Taj, Zehra Agha, Raheel Qamar, Jamal B. Williams, Maleeha Azam

**Affiliations:** 1 Translational Genomics Laboratory, Department of Biosciences, COMSATS University Islamabad, Park Road, Tarlai Kalan, Islamabad 45600, Pakistan; aisha.ncvi@gmail.com (A.N.H.); zehranajam@gmail.com (Z.A.); 2 Department of Psychiatry, Pakistan Institute of Medical Sciences, Islamabad 44000, Pakistan; rizwantajpims@gmail.com; 3 Department of Psychiatry, Jacobs School of Medicine and Biomedical Sciences, State University of New York at Buffalo, Buffalo, NY 14203, USA; 4 Pakistan Academy of Sciences, Islamabad 44000, Pakistan; drraheel@gmail.com; 5 Science and Environment Sector, Islamic World Educational, Scientific and Cultural Organization—ICESCO, Rabat 10104, Morocco

**Keywords:** monoamine oxidase, functional polymorphisms, psychiatric disorders, population genetics

## Abstract

**Background:** Monoamine oxidase A (MAOA) has a role in metabolising different biogenic amines, including dopamine. Functional studies have revealed the effect of promoter region variants on the transcriptional activity of the *MAOA* that consequently affects the homeostasis of the biogenic amines which might implicate in the aetiology of multiple psychiatric conditions. **Objectives:** The current study aimed to determine the influence of the promoter region 30 base pair (bp), a variable number of tandem repeats (VNTR) of the *MAOA*, on its serum levels and association with schizophrenia (SHZ), bipolar disorder (BD), and major depressive disorder (MDD) in the Pakistani population. **Methods:** A total of 1062 subjects [MDD *n* = 416, BD *n* = 200, SHZ *n* = 97 and controls *n* = 349], were genotyped for *MAOA*-30bp µVNTR through standard polymerase chain reaction technique and logistic regression was applied to determine the genetic association. Serum MAOA levels were determined through enzyme-linked immunosorbent assay (ELISA) and the Mann-Whitney U test was applied. **Results:** In genotype analysis, eight different repeat (R) alleles of *MAOA*-30 bp µVNTR were observed, where 4.5R, 5.5R, and 6R were the rare repeats found in the current Pakistani cohort. In serum-based analysis the total MAOA serum levels were found to be significantly elevated in SHZ; however, in sub-group analysis, significantly higher serum levels of MAOA were observed only in the rare allele groups of MDD, BD, and SHZ. **Conclusions:** The current study gives us further insights into the complex nature of *MAOA* regulation and its genetic and serum-levels association with different psychiatric conditions.

## 1. Introduction

Monoamine oxidase A (MAO-A) is a metabolizing enzyme encoded by the X-linked gene MAOA. The enzyme is located in the mitochondrial outer membrane and functions to degrade different biogenic amines, including neurotransmitters such as dopamine (DA), serotonin (5HT), and norepinephrine (NE), and regulates the levels of these biogenic amines in extra- and intra-cellular spaces [1]. Multiple neuropsychiatric conditions, such as schizophrenia (SHZ) and mood disorders, are associated with the dysfunction of metabolizing enzymes, including MAOA [2,3]. Studies have indicated the role of MAOA enzyme activity in the etiology of major depressive disorder (MDD) and suicidality) [1,4].

Various genetic variants of *MAOA* have been reported in association with multiple psychiatric conditions, specifically a variable number of tandem repeats (VNTR), *MAOA*-30 bp µVNTR (rs1346551029), in the promoter region. This 30 bp µVNTR was reported to be a potential risk-associated functional polymorphism, located 1.2 kb upstream of the start codon [5]. This functional polymorphism *MAOA*-30 bp µVNTR has been reported to be associated with bipolar disorder (BD), (Jacob et al., 2005) [6,7], SHZ [8], MDD [9], and impulsivity and aggressive behavior [10].

The *MAOA*-30 bp µVNTR has five different reported alleles (2R, 3R, 3.5R, 4R, and 5R), depending on the copies of the 30 bp tandem repeat sequence, which have also been associated with differences in MAOA expression, as the functional studies have revealed the effect of these reported alleles (2R, 3R, 3.5R, 4R, and 5R) on variable transcriptional activity of the *MAOA* promoter, leading to differential expression levels based on which these alleles are categorized as low-activity group (2R, 3R, and 5R) and high-activity group (3.5R and 4R) [11,12,13]. Studies have indicated that individuals with the low-functioning MAOA genotype exhibit a neural profile characterized by elevated emotional reactivity and impaired inhibitory control. The low-functioning MAOA genotype is associated with reduced levels of MAOA, leading to increased and dysregulated central serotonin levels [14,15]. In addition, research has also indicated a significant association of 3R with the increased risk of male adolescent criminal behavior when interacting with psychosocial factors [16].

Hence, the available data related to MAOA expression in mood disorders in other ethnicities are very limited, whereas the role of *MAOA*-30 bp µVNTR in common psychiatric conditions, including MDD, BD, and SHZ, in individuals of Pakistani descent has not been established yet; therefore, the current study aimed to investigate the genetic association of promoter region functional polymorphism (*MAOA*-30 bp µVNTR) with MDD, BD, and SHZ, and the effect of different repeat alleles on MAOA serum levels in cases compared to controls in the Pakistani population.

## 2. Materials and Methodology

### 2.1. Ethical Approval

The current study conforms to the Helsinki Declaration and was approved by the Ethics Review Board of the Department of Biosciences, COMSATS University Islamabad, Pakistan (CIIT/Bio/ERB/19/84, 25 March 2019). All the subjects were informed about the study objectives, and written consent was obtained from the subjects or the family member of the patient. All participants’ privacy rights were kept under consideration, and their identity was anonymized.

### 2.2. Inclusion Criteria

To be included in the study, the participants (cases and controls) could be male or female and had to be between the ages of 18 and 65 years; they also could not suffer from any metabolic disorders. Following the ICD-10 guidelines (Codes F20–F29 for SHZ and Codes F30–F39 for BD and MDD) [17], patients must fulfil the diagnostic criteria for the relevant psychiatric illness.

### 2.3. Exclusion Criteria

Any participant suffering from a thyroid disorder; malignancies; contagious infectious diseases such as HIV (Human Immune Deficiency Virus), HCV (Hepatitis C Virus), and MTB (Mycobacterium tuberculosis); or have any medical condition that potentially interferes with brain function were excluded from the studied cohort. Those participants were also excluded from the control group if they had a history of any psychiatric or neurological disorder, stroke, or serious brain injuries, or had a positive family history of psychiatric and neurological disorders.

### 2.4. Subjects Recruitment and Blood Sample Collection

The current study’s participants were recruited from the Pakistan Institute of Medical Sciences (PIMS) hospital in Islamabad, Pakistan. The participants were diagnosed according to the standard diagnostic guidelines and classified as explained in the DSM-V (Diagnostic and Statistical Manual of Mental Disorders—Version 5), published by the American Psychiatric Association (2013); in the International Classification of Diseases, Tenth Revision (ICD-10), by the World Health Organization (2019b); and by qualified registered psychiatrists of PIMS. The healthy controls were also recruited from PIMS and the university (COMSATS University Islamabad (CUI), Pakistan) by conducting a questionnaire-based interview. The psychiatrists confirmed whether or not participants had any mental illnesses.

The sampling was random, at the individual level, ensuring an unbiased selection process and enhancing the representativeness of our study population. In total, *n* = 1062 subjects, including MDD (*n* = 416), BD (*n* = 200), SHZ (*n* = 97), and healthy controls (*n* = 349), were recruited for this study. The descriptive characteristics of all cases and controls are summarized in Table 1. Approximately 5cc (~5 mL) of blood was drawn from each participant (cases and controls), of which 3cc blood was added to an ethylenediaminetetraacetic acid (EDTA) tube (1 Becton Drive, Franklin Lakes, NJ, USA) for genomic DNA extraction according to the standard phenol–chloroform DNA extraction protocol [18]. The remaining 2cc of blood was added to the gel activator-containing tubes (1 Becton Drive, Franklin Lakes, NJ, USA) for the separation of serum via centrifugation for 10 min at 2000× *g* at 4 °C. The supernatants (serum) were collected in sterile Eppendorf tubes and were stored at −40 °C till further use.

### 2.5. Genotyping

*MAOA* 30 bp VNTR (rs1346551029) was genotyped by standard polymerase chain reaction (PCR) using the following set of primers: forward primer, 5′TGCTCCAGAAACATGAGCAC 3′; and reverse primer, 5′TAGACTTGGGGATCCGACTG 3′. The PCR was performed using 5× FIREPOL^®^ Master Mix ready to load from the Solis BioDyne (Cat. No. 04-12-00115; Tartu, Estonia). The thermal profile for amplification consisted of initial denaturation at 95 °C for 6 min, followed by 35 cycles of 95 °C for 35 s, 58 °C for 45 s, 72 °C for 45 s, and a final extension at 72 °C for 10 min. The amplified (PCR products) fragments of different alleles were visualized on a 4% agarose gel. In addition to genotyping, for validation, Sanger sequencing of 10% of the total samples was also performed.

### 2.6. MAOA Serum Level Assessment

A subset from each case group (MDD, BD, and SHZ) and the controls was selected based on genotype. The selected samples of cases and controls were grouped based on allele activity—low-activity alleles (MAOA-LA = 2R, 3R, 5R), high-activity alleles (MAOA-HA = 3.5R, 4R), medium-activity alleles (MAOA-MA = 4R/5R), and rare alleles (MAOA-RA = 4.5R, 5.5R, 6R)—and subjected to serum-level quantification through enzyme-linked immunosorbent assay (ELISA). A human MAOA ELISA kit (Type A Monoamine Oxidase, ELAB science Cat. No. E-EL-H1157, Houston, TX, USA) was used as per the manufacturer’s instructions for the detection of MAOA levels in the serum samples. The optical density (OD) of each well was determined with a micro-ELISA plate reader (chemplate reader model: CPR-201; Medperlong; Beijing, China), which was set to 450 nm.

### 2.7. Statistical Analysis

All the statistical analyses were performed using R (v 4.0.0) [19]. Fisher’s *t*-test was applied for continuous variables, and the χ^2^ test was applied for categorical variables in both cases and controls. The genetic analysis of *MAOA*-µVNTR was conducted similarly to Kuepper et al.’s method (2013) [20], by categorizing alleles into groups based on activity. Univariate logistic regression analysis was applied to determine the association, followed by multivariate logistic regression analysis (adjusted for age). The ELISA-based serum levels were analyzed between phenotypes (MDD, BD, and SHZ) and controls using the unpaired, non-parametric Mann–Whitney *U* test (2-tailed); Kruskal–Walli’s ANOVA test; and Dunn’s multiple comparison testing (for multiple comparison testing between the groups), using GraphPad Prism (VII). The threshold significance was kept at a *p*-value ≤ 0.05 in all statistical tests; however, the *p*-values were adjusted after multiple testing, where required.

## 3. Results

### 3.1. Genetic Association of MAOA-30 bp µVNTR

Eight different repeat (R) alleles, 2R, 3R, 3.5R, 4R, 4.5R, 5R, 5.5R, and 6R, of 30 bp *MAOA*-µVNTR were observed, and 4.5R, 5.5R, and 6R were the rare repeat alleles found in the current study’s Pakistani cohort. The observed allele frequency of each observed allele in our current cohort is represented in Supplementary Table S1 (allele frequencies across disorders).

Thus, as per our analysis in the current cohort, the 4R allele showed the highest frequency, followed by the 5R allele, in males of each studied group (cases and controls). Meanwhile, in females, the genotype 4R/5R was found in higher frequency, followed by 4R, in each studied group (cases and controls). Given that the MAOA gene is located on chromosome X, the phenotype and genotype of *MAOA*-µVNTR may differ between females and males because of hemizygosity.

The observed alleles were categorized into groups based on their reported transcriptional activity into low-activity alleles (MAOA-LA = 2R, 3R, 5R) and high-activity alleles (MAOA-HA = 3.5R, 4R) [12,13]), and the genetic analysis of *MAOA*-µVNTR was conducted in the same way as that conducted by Kuepper et al. (2013). The rare alleles (4.5R, 5.5R, and 6R) were excluded from the statistical testing because of their lower frequency. The genotypic analysis showed no significant association of any of the observed alleles with the studied cohort (MDD, BD, and SHZ) in both univariate and multivariate analysis (*p* > 0.05; Table 2).

### 3.2. Serum-Based Analysis of MAOA Protein Concentration

**Figure 1:**
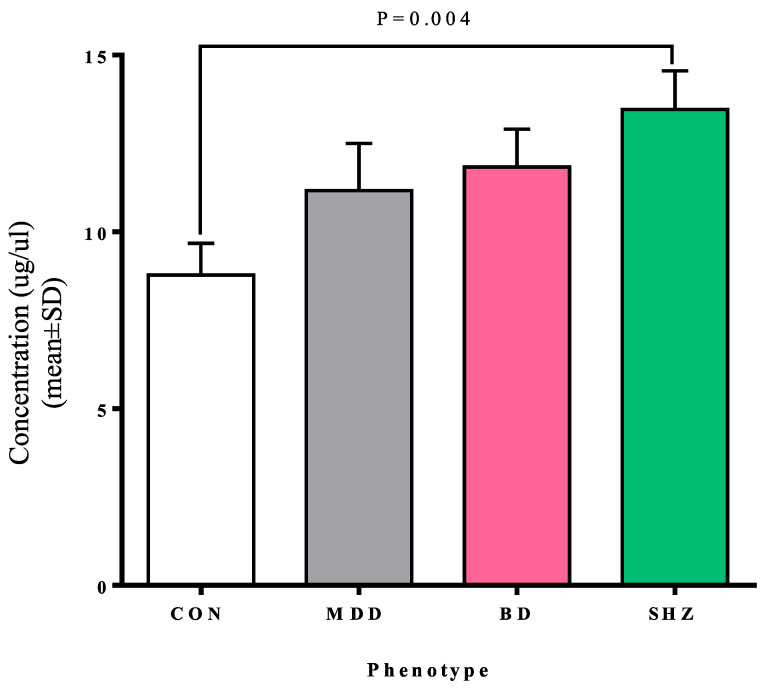
The bar chart represents overall serum MAOA concentration levels in each group. Y-axis, serum MAOA concentration; X-axis, phenotypes; CON, control; MDD, major depressive disorder; BD, bipolar disorder; SHZ, schizophrenia.

Descriptive features and the total serum concentrations of the selected samples (sub-cohort) from the studied cohort (cases and controls) are given in Table 2, and the descriptive statistics are in Supplementary Table S2. The serum levels of MAOA were found not to be normally distributed (Supplementary Figure S1). Therefore, a non-parametric Mann–Whitney U test was applied. In the initial analysis, we investigated the association of the total MAOA levels with the phenotypes compared to controls. The total MAOA serum levels were found to be elevated (*p* = 0.004) in the SHZ group as compared to the control group (Table 3 and Figure 1).

**Figure 2:**
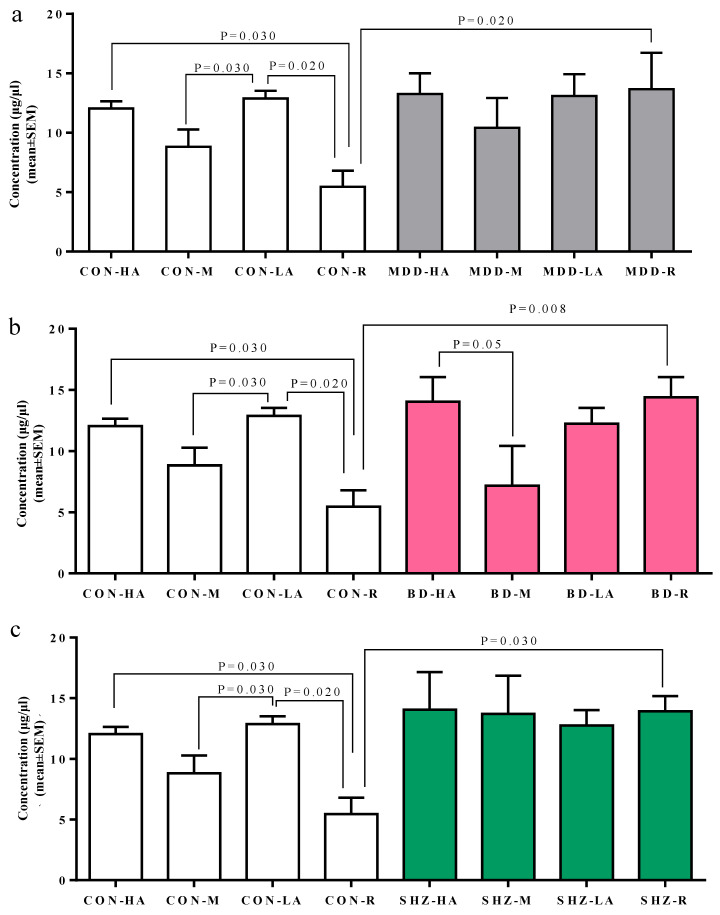
The bar chart represents overall serum MAOA concentration levels in each sub-group concerning alleles: (**a**) MDD (grey) compared to controls, (**b**) BD (pink) compared to controls, and (**c**) SHZ (green) compared to controls. Y-axis, serum MAOA concentration; X-axis, alleles; *p*-value from non-parametric Mann–Whitney *U* test. CON, control; MDD, major depressive disorder. CON-HA, MDD-HA, BD-HA, and SHZ-HA: high-activity alleles (3.5R and 4R). CON-LA, MDD-LA, BD-LA, and SHZ-LA: low-activity alleles (2R, 3R, and 5R). CON-M, MDD-M, BD-M, and SHZ-M: medium-activity alleles (4R/5R). CON-R, MDD-R, BD-R, and SHZ-R: rare alleles (4.5R, 5.5R, and 6R).

In the second analysis, the MAOA serum levels were analyzed within each group (cases and controls) based on genotype. The sub-cohorts of cases and controls were grouped based on groups of low-activity alleles (MAOA-LA = 2R, 3R, 5R), high-activity alleles (MAOA-HA = 3.5R, 4R), medium-activity alleles (MAOA-MA = 4R/5R), and rare alleles (MAOA-RA = 4.5R, 5.5R, 6R). Descriptive statistics of all the genotype-based selected sub-cohorts are represented in (Supplementary Table S3). Significant differences in serum MAOA concentration levels were observed within the control group (Figure 2). Significantly elevated levels of MAOA concentration were observed in higher-activity (CON-HA), lower-activity (CON-LA), and intermediate-activity (CON-M) alleles compared to the rare repeat allele group (CON-R) (*p* = 0.030, *p* = 0.020, and *p* = 0.030 respectively; Table 4).

However, in cross-group analysis, significant differences were observed only in the rare allele groups. Compared to controls (CON-R), higher levels of MAOA serum concentrations were observed in cases: MDD-R (*p* = 0.002), BD-R (*p* = 0.008), and SHZ-R (*p* = 0.03) (Figure 2 and Supplementary Table S4).

## 4. Discussion

The present study demonstrated the role of promoter region functional polymorphism (30 bp *MAOA* µVNTR in the etiology of multiple psychiatric disorders), and to the best of our knowledge, this current study on MAOA serum levels is the first to investigate and reveal the influence of 30 bp µVNTR in MAOA promoter on serum levels of MAOA in multiple psychiatric disorders in the Pakistani cohort. In the present study, genotype analysis showed that, in addition to previously reported repeats, 4.5R, 5.5R, and 6R were present, but comparatively in a lower frequency than 4R and 5R. A study by Al-Tayie et al. (2018) reported the same alleles (4.5R and 5.5R), in addition to 3.5R, in the Iraqi population [21]. However, in the current analysis, we did not find any potential association of the observed alleles with any of the psychiatric phenotypes (MDD, BD, and SHZ) in our Pakistani cohort. Similar non-significant findings have been observed in a BD family-based study in Italian and Canadian populations [22]. A study based on SHZ in Caucasians also revealed a negative association [23], whereas, in contrast to the present findings, a study in the Swedish population reported the association of low-activity alleles with SHZ in males but not in females [24]. Some studies on neuro-psychiatric conditions have also reported the association of low-activity MAOA alleles with impulsivity and aggression [10,24], and high-activity *MAOA* alleles with attention-deficient/hyperactivity disorder (ADHD) [25,26,27].

In addition to genetic association, the serum-based expression analysis was also conducted for the observed repeats of MAOA in the present study. Like the current analysis, comparative ELISA-based serum estimation of the MAOA levels affected by the presence of 30 bp µVNTR in the promoter region of MAOA was not observed before for MDD, BD, and SHZ in the Pakistani population. The available data on MAOA expression in these phenotypes in other ethnicities are very limited. However, the first functional study that reported the differential expression and activity of MAOA based on alleles (with different numbers of repeats) was through luciferase reporter gene fusions and the transfection experiments in cell lines of human neuroblastoma and placental choriocarcinoma [28].

According to the previous literature, alleles 3R and 4R are reported to be the most prevalent forms of repeats; however, the functional studies have revealed the association of 3R, 5R, and the 2R repeat alleles with 2–10-fold reduction in MAOA transcriptional activity in comparison to 4R and 3.5R [28,29,30]. In our study, we observed overall elevated serum MAOA levels in association with SHZ regardless of which repeat allele they carried. On the other hand, we observed that the rare repeat alleles (MAOA-RA = 4.5R, 5.5R, 6R) were found to have a significant association with higher expression of MAOA in all phenotypes (BD, MDD, and SHZ) in comparison to our control group; however, the serum MAOA levels in rare repeat allele carriers in controls were also found to be lower than MAOA-HA allele carriers within the control group.

Though MAOA µVNTR is one of the most studied variants concerning temperament and behavioral traits, some studies have revealed that this gene possesses another VNTR, which is approximately 500 bp upstream of the 30 bp µVNTR location and is termed as distal or dVNTR [31]. Studies have also reported the two coding transcripts for the MAOA gene and have also indicated a minimum of two transcriptional start sites (TSSs). One of the isoforms encompasses the 30 bp µVNTR within the 5′ UTR and was termed the primary MAOA isoform, which is also the most abundant transcript/mRNA of MAOA [31,32,33]. The functional study by [31] has determined the role of these VNTRs and that the expression of these 2 isoforms of the MAOA gene is differentially modulated by these two promoter regions’ VNTRs (µVNTR and dVNTR); thus, the effect of the combined expression profiling of these VNTRs offers new insights into the MAOA gene regulation, gene–environment interaction, and MAOA-driven behavioral traits [31]. However, most of the reported studies refer only to the 30 bp µVNTR as the major or sole regulator or the mediator of MAOA gene expression. It has also been considered to be a possible biomarker for certain stress-related illnesses based on different repeat alleles implicated in MDD, addiction, and violence [34,35,36,37,38]; in suicidality in BD females [39]; and in MDD males [1].

## 5. Conclusions

To conclude, this is the first report from the Pakistani population. Though we have focused on the µVNTR element only to account for variations in *MAOA* expression and serum levels and as a contributing risk factor to MDD, BD, and SHZ in our Pakistani cohort, the current study gives us further insights into the complex nature of *MAOA* regulation and its association with different psychiatric conditions. Nevertheless, the evidence we have provided is only a partial explanation regarding *MAOA* 30 bp µVNTR; the small cohort size is the limitation of the current study, as it is always very challenging with smaller cohort sizes to decide on a genetic-association study and ELISA-based expression analysis in multifactorial disorders due to the diversity of population genetics. Moreover, another reported dVNTR in the distal region of the promoter also has the potential to regulate MAOA expression. Therefore, the X-linked MAOA gene polymorphism association with MDD, BD, and SHZ is still inconclusive, which is more likely due to the sex- and phenotype-based differences, and also we cannot rule out the potential interactive contribution of the genotype/phenotype (different repeats allele and phenotype) of both VNTRs and the gene/environment to influence the expression of MAOA and its serum-based levels in different ethnicities. However, these approaches are not feasible at this stage, but we are actively working toward incorporating them into future research.

## Figures and Tables

**Table 1:** Descriptive statistics of the studied cohort.

Demographic Factors		CON	MDD	BD	SHZ	MDD vs. CON *p*	BD vs. CON *p*	SHZ vs. CON *p*
	Total (*n*)	355	428	206	131	NA	NA	NA
1	Age (years)	24.06 ± 6.11	32.93 ± 11.61	30.20 ± 11.22	29.68 ± 10.01	<2.2 × 10^−16^	4.15 × 10 ^12^	1.03 × 10^−8^
2	Age at onset (years)	NA	29.10 ± 11.12	23.57 ± 9.72	23.32 ± 8.87	NA	NA	NA
3	Sex (male) (female)	204 (57.0%) 151 (43.0%)	165 (39.0%) 263 (61.0%)	122 (59.0%) 84 (41.0%)	93 (71.0%) 38 (29.0%)	1.11 × 10^−7^ *	0.68 *	0.005 *
4	Ethnicity (Punjabi)	245 (69%)	288 (67.30%)	155 (75.00%)	103 (79.00%)	0.61 *	0.11 *	0.03 *
5	Tobacco users	22 (6.20%)	70 (16.35%)	56 (27.18%)	45 (34.35%)	4.64 × 10^−6^ *	1.60 × 10^−9^ *	1.27 × 10^−9^ *
6	Cannabis abusers	0	15 (3.50%)	18 (8.74%)	11 (8.40%)	NA	NA	NA

Values are *n* (%) or mean ± SD; *p*-value from Welch’s two-sample *t*-test, and * *p*-value from Pearson’s Chi-squared test χ^2^ with Yates’s continuity correction. NA, not applicable; CON, control; MDD, major depressive disorder; BD, bipolar disorder; SHZ, schizophrenia.

**Table 2:** Genetic association of *MAOA*-30 bp VNTR (rs1346551029).

Females										
*MAOA*-30 bp VNTR (rs1346551029)					Univariate Analysis			Multivariate Analysis *		
Phenotype					MDD vs. CON	BD vs. CON	SHZ vs. CON	MDD vs. CON	BD vs. CON	SHZ vs. CON
Genotype	Control *n* = 151	MDD *n* = 263	BD *n* = 84	SHZ *n* = 40	OR (95% CI) *p*	OR (95% CI) *p*	OR (95% CI) *p*	OR (95% CI) *p* *	OR (95% CI) *p* *	OR (95% CI) *p* *
HA	55 (36.42%)	76 (28.90%)	26 (30.95%)	9 (22.5%)	1.05 (0.98–1.12) 0.16	1.05 (0.96–1.1) 0.30	0.30 (0.98–1.15) 0.14	1.02 (0.96–1.08) 0.60	1.02 (0.94–1.11) 0.56	1.04 (0.96–1.13) 0.32
HA/LA	69 (45.70%)	133 (50.57%)	39 (46.43%)	22 (55.0%)						
LA	27 (17.88%)	54 (20.53%)	19 (22.62%)	9 (22.5%)						
**Males**										
***MAOA*-30 bp VNTR (rs1346551029)**					**Univariate Analysis**			Multivariate Analysis *		
**Phenotype**					**MDD vs. CON**	**BD vs. CON**	**SHZ vs. CON**	**MDD vs. CON**	**BD vs. CON**	**SHZ vs. CON**
**Genotype**	**Control** ***n* = 198**	**MDD** ***n* = 153**	**BD** ***n* = 116**	**SHZ** ***n* = 77**	**OR** **(95% CI) *p***	**OR** **(95% CI) *p***	**OR** **(95% CI) *p***	**OR** (95% CI) *p* *	**OR** (95% CI) *p* *	**OR** (95% CI) *p* *
HA	114 (57.58%)	83 (54.25%)	68 (58.62%)	45 (58.44%)	1.03 (0.93–1.15) 0.53	0.99 (0.89–1.10) 0.86	0.99 (0.89–1.10) 0.90	1.04 (0.95–1.15) 0.37	0.98 (0.88–1.08) 0.68	1.01 (0.91–1.12) 0.84
LA	84 (42.42%)	70 (45.75%)	48 (41.38%)	32 (41.56%)						

*p* (univariate analysis) and *p* * (multivariate analysis, adjusted for age) are from Chi-squared χ^2^ test with Yates’s continuity correction. OR, odds ratio; CI, confidence interval; HA, high-activity repeat (3.5R and 4R); LA, low-activity repeat (2R, 3R, and 5R).

**Table 3:** Descriptive features of subset cohort for MAOA ELISA.

Subjects	CON	MDD	BD	SHZ	MDD vs. CON	BD vs. CON	SHZ vs. CON
*n*	22	22	22	22	NA	NA	NA
Sex (male) (female)	8	8	11	13	1.00	0.54	0.23
Age (years) ** (Mean ± SD)	22.10 ± 2.40	30.04 ± 7.58	31.59 ± 12.4	34.54 ± 10.26	8.196 × 10^−5^	0.002	1.166 × 10^−5^
Age of onset (years) (Mean ± SD)	-	26.84 ± 8.29	24.41 ± 11.10	25.18 ± 9.47	-	-	-
Tobacco users	2 (9.09%)	4 (18.18%)	6 (27.27%)	8 (36.36%)	0.66	0.241	0.07
Suicidal	-	19 (86.36%)	18 (81.81%)	15 (68.18%)	-	-	-
Aggression	-	15 (68.18%)	15 (68.18%)	8 (36.36%)	-	-	-
Insomnia	-	13 (59.09%)	10 (45.45%)	8 (36.36%)	-	-	-
Ethnicity (Punjabis) *	11 (50.0%)	11 (50.0%)	15 (68.18%)	18 (81.81%)	1.00	0.36	0.06
Total serum MAOA ^#^ levels ng/µL (Mean ± SD)	8.78 ± 4.12	11.2 ± 6.12	11.8 ± 4.92	13.5 ± 4.89	0.159	0.061	0.004

Values in the table are given as *n* (%) or Mean ± SD; * *p*-value from Pearson’s Chi-squared χ^2^ test with Yates’s continuity correction; ** *p*-value from Welch’s two-sample *t*-test; ^#^ *p* valve from non-parametric Mann–Whitney *U* test; ng/µL, nanogram per microliter; CON, control; BD, bipolar disorder; SHZ, schizophrenia; MDD, major depressive disorder.

**Table 4:** Serum MAOA concentration level analysis within each group, concerning genotype.

Comparative Analysis	*p*-Value
CON-HA vs. CON-LA	0.48
CON-HA vs. CON-M	0.09
CON-HA vs. CON-R	**0.03**
CON-LA vs. CON-M	**0.03**
CON-LA vs. CON-R	**0.02**
CON-M vs. CON-R	0.28
MDD-HA vs. MDD-LA	0.85
MDD-HA vs. MDD-M	0.55
MDD-HA vs. MDD-R	0.83
MDD-LA vs. MDD-M	0.57
MDD-LA vs. MDD-R	0.76
MDD-M vs. MDD-R	0.51
BD-HA vs. BD-LA	0.43
BD-HA vs. BD-M	**0.05**
BD-HA vs. BD-R	0.94
BD-LA vs. BD-M	0.22
BD-LA vs. BD-R	0.52
BD-M vs. BD-R	0.19
SHZ-HA vs. SHZ-LA	0.43
SHZ-HA vs. SHZ-M	0.50
SHZ-HA vs. SHZ-R	0.68
SHZ-LA vs. SHZ-M	0.77
SHZ-LA vs. SHZ-R	0.49
SHZ-M vs. SHZ-R	0.70

CON, control; MDD, major depressive disorder. CON-HA, MDD-HA, BD-HA, and SHZ-HA: high-activity alleles (3.5R, 4R). CON-LA, MDD-LA, BD-LA, and SHZ-LA: low-activity alleles (2R, 3R, and 5R). CON-M, MDD-M, BD-M, and SHZ-M: medium-activity alleles (4R/5R). CON-R, MDD-R, BD-R, and SHZ-R: rare alleles (4.5R, 5.5R, and 6R). The significant values are underlined and bold.

## Data Availability

All the relevant data are mentioned in the manuscript, and the rest of the data are mentioned as Supplementary Data in an additional file, which will be available with the manuscript.

## Supplementary Materials

The following supporting information can be downloaded at https://www.mdpi.com/article/10.3390/biomedicines13030698/s1, Table S1. Allele Frequencies Across Disorders; Table S2. Descriptive statistics of serum MAOA concentration in each group; Table S3. Descriptive statistics of serum MAOA concentration in each group concerning genotype; Table S4. Serum MAOA concentration levels analysis across each group concerning genotype; Figure S1. The histogram represents the distribution of serum MAOA levels in 4 sub-cohorts. (a) CON: Controls; (b) MDD: Major depressive disorder; (c) BD: Bipolar disorder; (d) SHZ: Schizophrenia. The Y-axis represents the frequency (number of individuals), and the X-axis represents the MAOA concentration levels ng/µl.
