# Supplementary material for: Association of Monoamine Oxidase A Gene Promoter Region (30 bp μVNTR) Polymorphism with Serum Levels in Multiple Psychiatric Disorders
Source: Biomedicines. 2025 Mar 12;13(3):698. doi: 10.3390/biomedicines13030698 (PMC11940785; doi:10.3390/biomedicines13030698)
Supplement: Supplementary file 1 [file biomedicines-13-00698-s001.zip › biomedicines-3408256-supplementary.pdf]

Association of monoamine oxidase A gene promoter region (30bp μVNTR) polymorphism with serum levels in multiple psychiatric disorders

Aisha Nasir Hashmi[1], Rizwan Taj[2], Zehra Agha[1,3], Raheel Qamar[4, 5], Jamal B. Williams[3*] and Maleeha Azam[1*]

[1]Translational Genomics Laboratory, COMSATS University Islamabad, Pakistan

[2]Department of Psychiatry, Pakistan Institute of Medical Sciences, Islamabad, Pakistan

[3]Department of Psychiatry, Jacobs School of Medicine and Biomedical Sciences, State University of New York at Buffalo, Buffalo, NY 14203, USA

[4]Pakistan Academy of Sciences, Islamabad, Pakistan

[5]Science and Technology Sector, ICESCO, Rabat, Morocco

[*]Shared correspondence

Supplementary Table S1. Allele Frequencies Across Disorders

Disorder / Gender	n	3R	3.5R	4R	5R	3R/4R	4R/5R	4.5R	5.5R	4.5R/5.5R	4.5R/5R	5R/5.5R	6R	4R/6R	5R/6R	4R/5.5R	2R/5R
**MDD Female**	282	0.35%	0.35%	26.60%	18.79%	1.42%	45.74%	1.42%	1.42%	0.35%	1.42%	1.42%	0.35%	0.71%	0.71%	-	-
**MDD Male**	162	-	1.23%	50.00%	43.21%	-	-	3.70%	-	-	-	-	1.85%	-	-	-	-
**BD Female**	87	-	0.40%	10.10%	7.66%	-	15.72%	-	-	-	-	-	-	0.40%	1.21%	-	-
**BD Male**	126	0.83%	0.83%	55.37%	38.84%	-	-	4.13%	-	-	-	-	-	-	-	-	-
**SHZ Female**	42	-	-	21.42%	21.42%	-	52.38%	-	2.38%	-	-	-	-	-	2.38%	-	-
**SHZ Male**	85	-	-	52.94%	37.65%	-	-	4.70%	2.35%	-	-	-	2.35%	-	-	-	-
**CON Female**	160	0.62%	-	34.37%	15.63%	-	43.12%	1.89%	-	0.62%	-	0.62%	-	-	-	0.62%	0.62%
**CON Male**	204	0.49%	-	55.88%	40.20%	-	-	2.94%	-	-	-	-	0.49%	-	-	-	-

The table represent the percentage of observed allele frequency of each observed allele in our current cohort Eight different repeats (R) alleles, 2R, 3R, 3.5R, 4R, 4.5R, 5R, 5.5R, and 6R of 30bp MAOA-µVNTR were observed, where 4.5R, 5.5R, and 6R were the rare repeat alleles found in the current study Pakistani cohort. n (sample size), CON: Controls, BD: Bipolar Disorder; SHZ: Schizophrenia; MDD: Major Depressive Disorder.

Supplementary Table S2. Descriptive statistics of serum MAOA concentration in each group

	CON	MDD	BD	SHZ
**Number of values**	22	22	22	22
**Minimum**	1.37	1.74	2.26	5.35
**25% Percentile**	4.88	5.21	8.98	9.23
**Median**	10.7	12.2	12	14
**75% Percentile**	11.9	15.7	15.6	17.1
**Maximum**	14.2	24.7	20.1	23.9
**Mean**	8.78	11.2	11.8	13.5
**Std. Deviation**	4.12	6.12	4.92	4.89
**Std. Error of Mean**	0.899	1.34	1.07	1.09
**Lower 95% CI of mean**	6.91	8.38	9.6	11.2
**Upper 95% CI of mean**	10.7	14	14.1	15.8
**Sum**	184	235	249	269

Supplementary Table S3. Descriptive statistics of serum MAOA concentration in each group concerning genotype

	CON-HA	CON-M	CON-LA	CON-R	MDD-HA	MDD-M	MDD-LA	MDD-R	BD-HA	BD-M	BD-LA	BD-R	SHZ-HA	SHZ-M	SHZ-LA	SHZ-R
Number of values	6	5	4	5	7	5	4	6	5	3	9	5	7	4	8	3
Minimum	11.05	4.31	11.06	3.19	9.73	4.52	9.79	4.94	9.58	3.94	8.31	9.52	6.39	7.45	8.32	11.54
25% Percentile	11.12	5.44	11.54	3.75	10.36	5.71	9.79	5.34	10.07	3.94	8.43	11.60	8.69	7.45	9.23	11.54
Median	11.68	10.71	13.11	6.69	12.60	8.83	13.31	14.63	12.58	7.18	11.99	13.90	18.28	16.33	13.18	14.38
75% Percentile	13.32	11.29	13.96	8.655	16.80	15.95	16.15	19.40	18.72	10.42	16.12	17.47	21.67	17.37	14.54	15.85
Maximum	13.76	11.84	14.2	8.89	18.08	18.53	16.15	24.66	20.15	10.42	16.29	19.87	23.89	17.37	19.33	15.85
**Mean**	**12.04**	**8.83**	**12.87**	**6.37**	**13.25**	**10.43**	**13.09**	**13.66**	**14.03**	**7.18**	**12.23**	**14.41**	**15.80**	**13.72**	**12.75**	**13.92**
**Std. Deviation**	**1.21**	**3.23**	**1.31**	**2.57**	**3.50**	**5.57**	**3.18**	**7.51**	**4.53**	**4.58**	**3.45**	**3.70**	**7.00**	**5.45**	**3.60**	**2.19**
**Std. Error of Mean**	**0.61**	**1.44**	**0.65**	**1.28**	**1.75**	**2.49**	**1.84**	**3.06**	**2.02**	**3.24**	**1.31**	**1.66**	**3.13**	**3.15**	**1.27**	**1.26**
Lower 95% CI of mean	10.11	4.82	10.78	2.28	7.67	3.51	5.18	5.77	8.41	-34	9.03	9.80	7.10	0.17	9.74	8.48
Upper 95% CI of mean	13.98	12.84	14.96	10.45	18.83	17.35	20.99	21.55	19.66	48.37	15.43	19.01	24.51	27.26	15.77	19.36
Sum	48.17	44.16	51.49	25.47	53.01	52.14	39.26	81.99	70.17	14.37	85.64	72.04	79.02	41.15	102	41.77

Supplementary Table S4. Serum MAOA concentration levels analysis across each group concerning genotype

Comparative Analysis	P value
CON-HA vs. MDD-HA	0.66
CON-HA vs. BD-HA	0.82
CON-HA vs. SHZ-HA	0.68
CON-LA vs. MDD-LA	0.85
CON-LA vs. BD-LA	0.91
CON-LA vs. SHZ-LA	>0.99
CON-M vs. MDD-M	0.77
CON-M vs. BD-M	0.28
CON-M vs. SHZ-M	0.25
CON-R vs. MDD-R	***0.002***
CON-R vs. BD-R	***0.008***
CON-R vs. SHZ-R	***0.03***
**Comparative Analysis**	**P value**
MDD-HA vs. BD-HA	> 0.99
MDD-HA vs. SHZ-HA	0.52
BD-HA vs. SHZ-HA	0.66
MDD-LA vs. BD-LA	0.83
MDD-LA vs. SHZ-LA	0.92
BD-LA vs. SHZ-LA	0.83
MDD-M vs. BD-M	0.38
MDD-M vs. SHZ-M	0.57
BD-M vs. SHZ-M	0.40
MDD-R vs. BD-R	0.87
MDD-R vs. SHZ-R	> 0.99
BD-R vs. SHZ-R	> 0.99

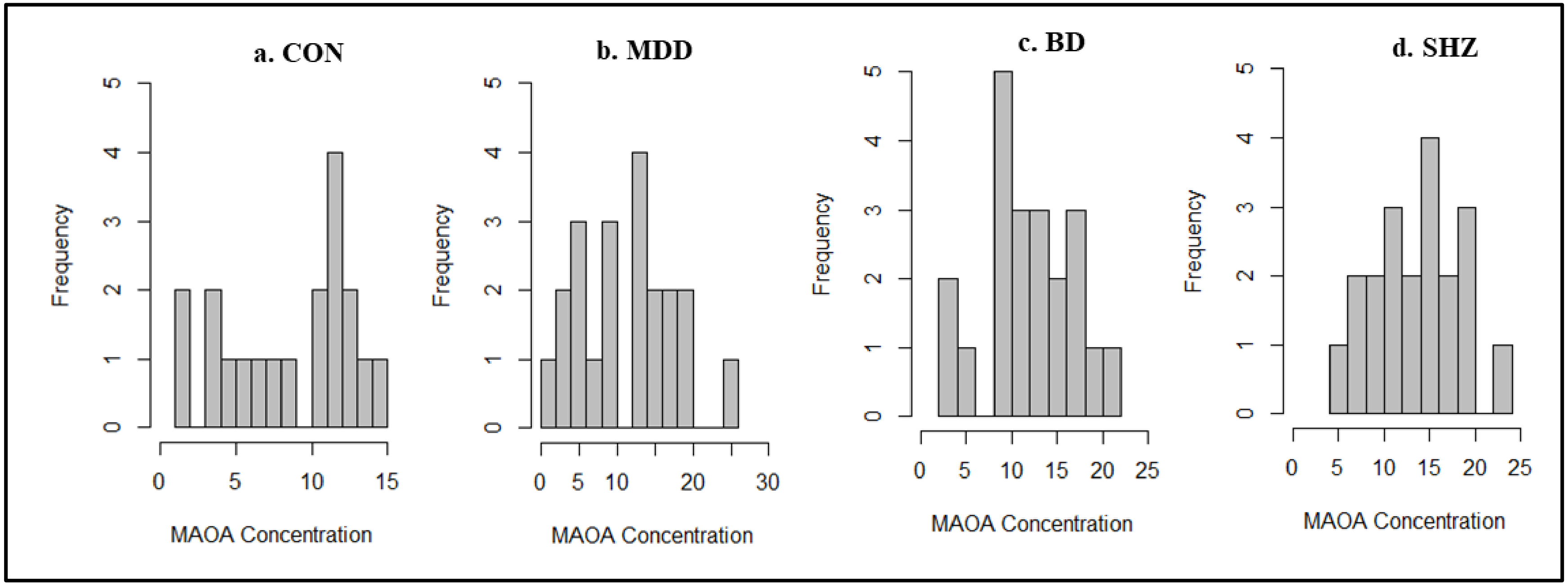

Supplementary Figure S1. The histogram represents the distribution of serum MAOA levels in 4 sub-cohorts. (a) CON: Controls; (b) MDD: Major depressive disorder; (c) BD: Bipolar disorder; (d) SHZ: Schizophrenia. The Y-axis represents the frequency (number of individuals), and the X-axis represents the MAOA concentration levels ng/µl.
